# The Effects of Boron Mineral on Performance, Bone and Mineral Metabolism in Purebred Arabian Foals

**DOI:** 10.1007/s12011-026-05138-x

**Published:** 2026-05-06

**Authors:** İsmail Çağrı Yilmaz, Talat Güler, Edanur Güler Ekmen, Mehtap Özçelik

**Affiliations:** 1 Sultansuyu Agricultural Enterprise, Malatya, Türkiye; 2 Department of Animal Nutrition, Veterinary Faculty of Fırat University, Elazığ, Türkiye; 3 Department of Physiology, Veterinary Faculty of Fırat University, Elazığ, Türkiye; 4 Vocational School of Health Services, Fırat University, Elazığ, Türkiye

**Keywords:** Boron, Foals, Performance, Mineral metabolism

## Abstract

Boron plays significant roles in various biological systems, including mineral, lipid, and energy metabolism, immune and endocrine systems, and brain function. It has been suggested to enhance performance and may prevent conditions such as osteoporosis, osteoarthritis, and arthritis. Despite these known benefits, its effects on growth performance and mineral metabolism in horses remain understudied, particularly in young animals like Purebred Arabian foals. The primary objective of this study was to investigate the effects of different doses of boron supplementation (0, 5, 10, and 15 mg/day per animal) on the performance (live weight gain, feed intake, feed conversion ratio) and bone and mineral metabolism in Purebred Arabian foals. A total of 32 Purebred Arabian foals with similar initial live weights were randomly divided into four groups, each consisting of eight animals. The experimental groups were as follows: Control Group (K Group): No boron supplementation. B5 Group: Received 5 mg/day of elemental boron. B10 Group: Received 10 mg/day of elemental boron. B15 Group: Received 15 mg/day of elemental boron. Boric acid was used as the boron source, and the study spanned 90 days. Feed intake, live weight gain, and feed conversion ratio were monitored. Measurements of metacarpal diameter, withers height, and chest circumference were recorded. Serum samples were analyzed for ALP, P, Mg, Ca, B, PTH, cortisol, calcitonin, osteocalcin, and vitamin D3 levels. Performance: Feed intake was similar across all groups (*P* > 0.05). However, the B15 group exhibited the highest live weight gain, daily weight gain, and feed conversion efficiency (*P* < 0.05). Bone and Mineral Metabolism: Serum levels of ALP, P, and Mg showed no significant differences between groups (*P* > 0.05). While no significant main effect of group was found for metacarpal diameter (*P* > 0.05), a highly significant time × group interaction was detected (*P* < 0.001), indicating that boron supplementation modulated the developmental trajectory of the skeleton. Serum calcium (Ca) levels on day 90 were significantly higher in the B15 group compared to the control group (P = 0.03). Serum boron (B) levels increased linearly with the administered dose (*P* < 0.001). Serum cortisol levels on day 90 were significantly higher in the B10 and B15 groups compared to the control (P = 0.004). Calcitonin and osteocalcin levels showed a dose-dependent linear increase, peaking in the B15 group on day 90 (*P* < 0.001). Vitamin D3 and PTH levels did not differ significantly among the groups (*P* > 0.05). Boron supplementation positively influenced growth performance and bone and mineral metabolism in Purebred Arabian foals, with the 15 mg/day dose showing the most pronounced benefits. These findings suggest that boron supplementation could be an a promising nutritional strategy to enhance growth performance and mineral metabolism in young horses. Further research is warranted to explore long-term effects and potential applications in equine nutrition and management. However, further long-term studies are required to confirm these findings and determine optimal supplementation levels.

## Introduction

Boron (B) is a trace mineral whose physiological importance has gained increasing attention due to its regulatory role in mineral metabolism, bone development, and endocrine function in animals [1–3]. Rather than serving as a structural component, boron influences the bioavailability and utilization of essential macro-minerals, particularly calcium (Ca), phosphorus (P), and magnesium (Mg), which are critical for skeletal integrity and growth [4, 5]. In addition, boron has been reported to interact with vitamin D metabolism and hormones involved in bone turnover, suggesting a role in maintaining skeletal health during periods of rapid growth and increased metabolic demand [6–8].

In horses, mineral balance is of particular importance because the skeletal system is subjected to substantial mechanical loading during growth and athletic training. Developmental orthopedic disorders in young horses are frequently associated with disruptions or imbalances in Ca, P, and Mg metabolism [6–9]. Recent equine nutrition studies emphasize that macro- and trace minerals function within a complex regulatory network that influences bone development, metabolic adaptation, and athletic performance [10, 11]. Investigations assessing mineral profiles in horses have shown that, under normal feeding conditions, blood mineral concentrations generally remain within physiological reference ranges; however, subtle variations may reflect adaptive metabolic regulation rather than toxicological risk, especially in athletic or growing horses [12, 13].

Limited but emerging evidence suggests that boron is metabolically active in horses and may influence mineral-related physiological responses. Oral supplementation of magnesium combined with boron has been shown to increase circulating ionized and total magnesium concentrations and to improve clinical outcomes in horses, indicating a potential synergistic interaction between boron and magnesium in equine mineral metabolism [12]. These findings support the hypothesis that boron may modulate mineral bioavailability and systemic physiological responses in horses, even when administered at nutritional supplementation levels.

In addition to supplementation studies, investigations evaluating trace mineral bioaccumulation in horses have demonstrated that elements such as boron, zinc, chromium, iron, and other trace minerals can be detected in blood and biological substrates, including mane and tail hair [14]. Associations between trace mineral concentrations and hematological parameters have been reported, suggesting that these elements participate in systemic metabolic processes and may reflect physiological adaptation rather than overt toxicity [13]. Such findings further emphasize the importance of understanding dose-dependent responses and mineral interactions in horses, particularly those exposed to increased metabolic demands.

Adequate nutrition during the growth period is essential for ensuring optimal performance and a healthy athletic career in adulthood. Diets provided to growing horses must be carefully balanced in energy, protein, and essential macro-minerals—especially Ca, P, and Mg—to support proper skeletal development and bone strength [6–9]. Horses bred for racing are particularly sensitive to nutritional imbalances during early growth, as skeletal maturation must coincide with increasing mechanical load and training intensity. Purebred Arabian foals represent a unique population in this context due to their early athletic expectations, high metabolic demands, and susceptibility to mineral imbalances during growth.

Despite growing interest in equine mineral nutrition, data regarding boron supplementation in horses remain limited. In particular, information on the effects of dietary boron on growth performance, bone metabolism, and mineral homeostasis during the growth period is scarce. Therefore, the present study aimed to investigate the effects of dietary boron supplementation on growth performance parameters, including live weight gain (LWG), feed intake, and feed conversion ratio (FCR), as well as on bone and mineral metabolism in Purebred Arabian foals. We hypothesized that appropriate boron supplementation would positively influence mineral metabolism and skeletal-related parameters without adversely affecting growth performance.

## Materials and Methods

### Animals and Feed Materials

The experiment was conducted at the Horse Unit of the Sultansuyu Agricultural Enterprise in Malatya. A total of 32 male Purebred Arabian foals, aged 6 months (mean ± SD: 180.0 ± 5 days; range: 175–185 days), were used in the study. All foals had been weaned at approximately 5 months of age and had completed a minimum 4-week post-weaning adaptation period before the start of the experiment, during which they were fed the same basal diet used in the trial. The feed materials used in the experiment consisted of meadow hay (*Bromus inermis*, *Lolium perenne*,* Poa pratensis*,* Dactylis*,* Glomerata*, *Agropyron cristatum*) (as roughage) and oats (*Avena sativa*), which were obtained from the same enterprise, and compound feed (25% crude protein, 2700 Kcal ME), which was sourced from Gözlü Agricultural Enterprise Directorate. All foals were housed and fed individually, and the ration was offered individually to each foal at the same time of day, allowing accurate monitoring of feed intake and refusals. Water was provided ad libitum. As a boron mineral source in the experiment, “Boric Acid” was used [15–17]. Boric acid was obtained from the National Boron Research Institute (BOREN, Ankara, Türkiye). The experiment lasted for 90 days. During the study, no structured exercise or conditioning program was applied, and foals were allowed free movement in an open paddock under uniform management conditions. The study was approved by the Local Animal Ethics Committee of Inönü University (Decision No: 2021/26-2−13092).

The composition of the compound feed used in the experiment is provided in Table 1, the amounts and proportions of roughage and concentrate feed given to the animals are presented in Table 2, and the nutrient content of the feeds and ration is shown in Table 3.

**Table 1:** Composition of the concentrate feed

Feeds	%
Oats	66
Barley	11,9
Wheat Bran	3,4
DDGS	2,38
Corn	3,4
Soybean meal	10,2
Vegetable oil	0,34
Salt	0,41
Limestone	0,68
Dicalcium Phosphate	0,34
Mycotoxin Binder	0,13
Molasses	0,68
Trace Minerals*	0,14

*: Per 1 kg of compound feed: 4,200,000 IU of vitamin A, 1,400,000 IU of vitamin D3, 10,500 mg of vitamin E, 180,000 mg of niacin, 75,000 mg of choline, 20,000 mg of manganese, 25,000 mg of iron, 25,000 mg of zinc, 5,000 mg of copper, 50 mg of cobalt, 200 mg of iodine, 180 mg of selenium

**Table 2:** Amounts and proportions of roughage and concentrate feed given to the animals

Days	Roughage Kg %		Concentrate Feed Kg %	
0–30. days	6.00	71.43	2.40	28.57
30–60. days	6.50	71.43	2.60	28.57
60–90. days	6.75	71.43	2.70	28.57

**Table 3:** The nutrient values of the feeds and ration

Dry matter, %	Roughage	Concentrate Feed	Ration
	94,09	92,33	93,58
Crude protein, %	14,41	16,28	14,94
Ash, %	10,36	4,14	8,58
Crude fat, %	2,63	3,49	2,87
Crude fiber, %	25,28	10,46	21,04
Energy, ME, kcal/kg*	2300	2727	2422
Boron, %	0.0029	0.0030	0.0029

*: It was obtained through calculation

## Method

### Experimental Design

The study was conducted on four experimental groups (*n* = 8 foals per group). The foals were selected from male Purebred Arabian foals born within an approximately 10-day period at the same breeding farm and had comparable birth weights. At the start of the experiment, foals were 180 ± 5 days of age, and their body weights were recorded at the study site (Taralsa, Türkiye). Following stratification based on initial body weight to ensure comparable mean body weights among groups, foals were randomly allocated to the experimental groups. The experimental groups were formed according to different doses of dietary boron supplementation.

The control group received no boron supplementation, while the B5, B10, and B15 groups received 5 mg, 10 mg, and 15 mg of elemental boron per day, respectively [18–20]. The selected boron doses were based on previous experimental studies conducted in horses and other mammals, which reported physiological effects without evidence of toxicity, and were chosen to evaluate a dose–response relationship rather than body weight–adjusted dosing. Boric acid was diluted in 10 mL of distilled water and administered orally once daily at 3:00 PM using a feeding syringe.

## Measurements and Analyses

### Live Body Weight (LBW) and Daily Weight Gain (DWG), Feed Intake, and Feed Conversion Ratio (FCR)

At the beginning of the experiment, the foals were weighed using a calibrated scale with a sensitivity of 0.5 kg to determine their initial body weight. The groups were then distributed so that the average live body weights were similar across groups, and the experiment began. The foals were weighed again in the morning before feeding on days 30, 60, and 90, and the live body weight (LBW) was regularly recorded. The daily live weight gain was calculated by dividing the difference between two consecutive weight measurements by the number of days between them.

Feed intake was recorded on an individual basis by weighing the offered feed and the refusals daily. Both concentrate and roughage were offered in predetermined amounts according to age-related nutritional requirements, and refusals were recorded individually to calculate actual intake.

The feed conversion ratio was determined by dividing the daily feed intake by the daily live weight gain.

### Bone Development, Withers Height, and Chest Circumference Measurements

To assess bone development in the foals, individual measurements were taken at the start of the trial, and on days 30, 60, and 90. The bone development was evaluated by measuring the diameter of the middle section of the left forelimb metacarpal bone. Additionally, on the same days, withers height and chest circumference were measured individually and recorded for each foal.

### Determination of the Nutrient Composition of the Roughage and Concentrate Feed

The determination of the nutrient composition of the roughage and concentrate feeds was carried out at the Animal Nutrition and Nutritional Diseases Department Laboratory, Faculty of Veterinary Medicine, Fırat University. The dry matter, ash, crude protein, and crude fat levels of the feeds were determined according to the analytical methods reported by AOAC [21], and the crude fiber level was determined according to Crampton and Maynard [22]. The energy content of the feeds was calculated using the ration program (At_V5.05) developed by Coşkun et al. [23]. The boron content of the roughage and concentrate feeds was determined at the Boron Research Institute (BOREN) under the Turkish Atomic Energy Authority, using adapted internal methods (EPA 3051 A and 6010 D methods).

### Determination of Blood Parameters

At the beginning of the trial, and on days 30, 60, and 90, Blood samples were collected from the jugular vein by an experienced veterinarian, using vacuum serum tubes without anticoagulant, in the morning before feeding and approximately 18 h after the last boron administration. Foals were accustomed to human handling as part of routine farm management, and blood sampling was performed with minimal restraint to reduce stress. The rationale for selecting biochemical and hormonal parameters (Ca, P, Mg, ALP, PTH, calcitonin, osteocalcin, vitamin D3) was their central role in bone metabolism and mineral homeostasis, which are directly relevant to the hypothesized effects of boron supplementation.

The collected blood samples were centrifuged at 3500 rpm for 15 min to obtain serum samples. Biochemical analyses for serum alkaline phosphatase (ALP), parathyroid hormone (PTH), calcium (Ca), magnesium (Mg), and phosphorus (P) were performed using a biochemical analyzer (ADVIA 1800-SIEMENS) at the Department of Biochemistry, Faculty of Medicine, Fırat University. Serum boron levels were determined at the Boron Research Institute (BOREN), under the Turkish Atomic Energy Authority, using internal methods (CEM//Mars6 microwave and Thermo Scientific/ICAP 7000 ICP OES operating instructions). Serum vitamin D_3_ (Kit: Vitamin D_3_ Universal, Method: ELISA, Device: Microplate reader: BIO-TEK EL X 800-Auto strip washer: BIO-TEK EL X 50), calcitonin (Kit: Calcitonin (CALCA) Horse, Method: ELISA, Device: Microplate reader: BIO-TEK EL X 800-Auto strip washer: BIO-TEK EL X 50), and osteocalcin (Kit: Osteocalcin (BGLAP) Horse, Method: ELISA, Device: Microplate reader: BIO-TEK EL X 800-Auto strip washer: BIO-TEK EL X 50) analyses were carried out through service acquisition from a specialized company (BARAN MEDİKAL, Ankara, Türkiye).

### Statistical Analyses

In this study, the sample size was determined using the G*Power software package (Version 3.1.9.2) with an effect size of 0.67, an alpha error of 0.05, and a power of 85%, resulting in a total of 32 foals (*n* = 8 per group, 4 groups) [24]. Data analysis was performed using the SPSS statistical software package (IBM SPSS Version 22.0) [25]. For data evaluation, the normality assumption of the data was tested using the “Shapiro-Wilk” test, and homogeneity of variances was assessed with the “Levene” test. When parametric test assumptions were met, one-way analysis of variance (ANOVA) was used to determine group differences, with the “Tukey” test used for post-hoc analysis in the case of homogeneous groups, and the “Tamhane T2” test for post-hoc analysis in heterogeneous groups. A two-way repeated measures analysis of variance (ANOVA) was performed to evaluate the effects of group (0,5,10,15 mg/kg), time (0,30,60,90), and their interaction (group × time). When the assumption of sphericity was violated, the Greenhouse–Geisser correction was applied. When significant main or interaction effects were detected, pairwise comparisons were conducted using the Least Significant Difference (LSD) test. Statistical significance was considered at *P* < 0.05.

## Results

In the study, the effects of boron administered in different doses on growth performance parameters such as feed intake, weight gain, feed conversion ratio, and measurements of metacarpal diameter, withers height, and chest circumference were examined in purebred Arabian foals. Additionally, the impact of boron on bone metabolism parameters including serum Ca, P, Mg, ALP, parathyroid hormone, calcitonin, osteocalcin, and vitamin D_3_ levels was investigated. The findings obtained are presented below.

**Table 4:** The Effect of Different Doses of Boron on Feed Intake, Weight Gain, Daily Weight Gain (DWG), and Feed Conversion Ratio (FCR) in Animals (*n* = 8)

		Groups					
	Days	Control	B5	B10	B15	*P*	Linear
Feed intake, kg	0–30	8,4 kg	8,4 kg	8,4 kg	8,4 kg	> 0.05*	> 0.05
	30–60	9,1 kg	9,1 kg	9,1 kg	9,1 kg	> 0.05*	> 0.05
	60–90	9,45 kg	9,45 kg	9,45 kg	9,45 kg	> 0.05*	> 0.05
Live weight, kg	0	239,3 ± 3,3^D^	238,4 ± 5,9^D^	239,2 ± 4,3^D^	237,3 ± 6,2^D^	> 0.05	> 0.05
	30	258,5 ± 4,1^C^	259,0 ± 4,9^C^	261,9 ± 3,9^C^	260,8 ± 5,4^C^	> 0.05	> 0.05
	60	268,8 ± 3,1^B^	268,9 ± 5,1^B^	273,9 ± 4,6^B^	274,8 ± 5,3^B^	> 0.05	> 0.05
	90	284,5 ± 3,5^A^	284,5 ± 5,8^A^	288,1 ± 5,1^A^	288,4 ± 6,0^A^	> 0.05	> 0.05
	RM	< 0.001	< 0.001	< 0.001**	< 0.001		
	Two-Way RM						
	Time	< 0.001					
	Group	> 0.05					
	TimeXGroup	> 0.05					
DWG, gr	0–30	640,0 ± 51,7^b^	686,6,±50,9^b^	756,6 ± 57,1^a^	783,3 ± 71,6^a^	0,030*	< 0.05
	30–60	343,3 ± 21,9^b^	330,0 ± 23,4^b^	400,0 ± 51,5^ab^	466,6 ± 40,7^a^	0,026*	< 0.05
	60–90	523,3 ± 32,6^a^	520,0 ± 33,8^a^	473,3 ± 30,7^b^	453,3 ± 26,1^b^	0,032*	< 0.05
	0–90	502,2 ± 10,8^b^	512,2 ± 18,6^b^	543,3 ± 31,4^ab^	567,7 ± 21,8^a^	0,013*	0.007
FCR	0–30	13,1 ± 1,6^a^	12,2 ± 1,2^a^	11,1 ± 1,4^ab^	10,7 ± 1,5^b^	0,041*	< 0.05
	30–60	26,5 ± 1,7^a^	27,6 ± 1,2^a^	22,7 ± 1,4^ab^	19,5 ± 1,4^b^	0,022*	< 0.05
	60–90	18,0 ± 1,3	18,1 ± 1,3	19,9 ± 1,3	20,8 ± 0,8	> 0.05*	> 0.05
	0–90	19,2 ± 0,4^a^	19,3 ± 0,6^a^	17,9 ± 0,8^ab^	17,0 ± 0,5^b^	0,038*	0.024

The data are presented as mean ± standard error

Data were analyzed using a two-way repeated-measures ANOVA to evaluate the effects of group, time, and their interaction (LSD, *p* < 0.05)

*One-way ANOVA was conducted, and Tukey’s test was used as a post hoc analysis for intergroup comparisons

Different lowercase letters (a-d) above the groups indicate significant differences between the means within the same row

Differences between measurement times were analyzed using repeated measures (RM) analysis

Different capital letters (A-D) above the groups indicate significant differences between the means in the same column

** Greenhouse-Geisser

-While time had a significant effect on CA (*p* < 0.001), the time × group interaction was not statistically significant (*p* > 0.05)

Daily feed consumption was similar across all groups (*P* > 0.05). The highest daily weight gain was observed in the B15 group, followed by B10, B5, and control groups (*P* = 0.013). No statistical difference in body weight was found between the groups at individual time points (*P* > 0.05), but a significant increase was observed over time in all groups, reflecting normal growth progression (*P* < 0.001). These findings clearly demonstrate a dose-dependent improvement in growth efficiency associated with boron supplementation. When examining daily weight gains in the groups, the highest average daily weight gain was recorded in the B15 group, followed by the B10, B5, and control groups (*P* = 0.013). Throughout the total study period, the most efficient feed conversion ratio was observed in the B15 group, followed by B10, B5, and control groups (*P* = 0.038).

**Table 5:** The effect of different doses of boron given to the animals on metacarpal diameter, withers height, and chest circumference (*n* = 8)

		Groups					
	Days	Control	B5	B10	B15	*P*	Linear
Metacarpal diameter, cm	0	17,4 ± 0,2^B^	17,0 ± 0,3^B^	17,0 ± 0,2^C^	16,8 ± 0,2^D^	> 0.05	> 0.05
	30	17,6 ± 0,2^AB^	17,7 ± 0,2^A^	17,3 ± 0,2^B^	17,3 ± 0,1^C^	> 0.05	> 0.05
	60	17,6 ± 0,1^A^	17,9 ± 0,2^A^	17,6 ± 0,2^A^	17,6 ± 0,1^B^	> 0.05	> 0.05
	90	17,8 ± 0,1^A^	17,9 ± 0,2^A^	17,6 ± 0,2^A^	17,8 ± 0,1^A^	> 0.05	> 0.05
	RM	0.005	< 0.001	< 0.001	< 0.001		
	Two-Way RM						
	Time	< 0.001					
	Group	> 0.05					
	TimeXGroup	< 0.001					
Withers height, cm	0	133,3 ± 1,3^D^	133,4 ± 1,3^D^	134,4 ± 1,2^D^	132,9 ± 1,0^D^	> 0.05	> 0.05
	30	135,6 ± 0,8^C^	136,0 ± 1,0^C^	137,1 ± 0,8^C^	136,1 ± 0,9^C^	> 0.05	> 0.05
	60	137,1 ± 0,8^B^	136,9 ± 1,0^B^	138,4 ± 0,8^B^	137,4 ± 0,8^B^	> 0.05	> 0.05
	90	140,4 ± 1,0^A^	140,1 ± 0,8^A^	141,4 ± 0,5^A^	141,4 ± 0,5^A^	> 0.05	> 0.05
	RM	< 0.001	< 0.001	< 0.001**	< 0.001		
	Two-Way RM						
	Time	< 0.001					
	Group	> 0.05					
	TimeXGroup	> 0.05					
Chest circumference, cm	0	141,8 ± 0,9^D^	141,3 ± 2,2^D^	141,9 ± 1,6^D^	140,0 ± 1,8^D^	> 0.05	> 0.05
	30	145,3 ± 0,7^C^	145,0 ± 1,7^C^	146,0 ± 1,2^C^	145,5 ± 1,6^C^	> 0.05	> 0.05
	60	147,1 ± 0,8^B^	147,4 ± 1,6^B^	148,1 ± 1,3^B^	148,5 ± 1,5^B^	> 0.05	> 0.05
	90	149,4 ± 0,6^A^	150,5 ± 1,6 ^A^	151,1 ± 1,1^A^	151,3 ± 1,3^A^	> 0.05	> 0.05
	RM	< 0.001	< 0.001	< 0.001	< 0.001**		
	Two-Way RM						
	Time	< 0.001					
	Group	> 0.05					
	TimeXGroup	> 0.05					

The data are presented as mean ± standard error

Data were analyzed using a two-way repeated-measures ANOVA to evaluate the effects of group, time, and their interaction (LSD, *p* < 0.05)

Differences between measurement times were analyzed using repeated measures (RM) analysis

Different capital letters (A-D) above the groups indicate significant differences between the means in the same column

**Greenhouse-Geisser

- Time showed a significant effect on all measurements reported in Table 5 (*p* < 0.001). However, the time × group interaction was significant only for metacarpal diameter (*p* < 0.001)

No statistical difference was observed between the groups in terms of metacarpal diameter, withers height, and chest circumference measurements (*P* > 0.05). However, a significant increase over time was observed in all groups (*P* < 0.001), reflecting normal skeletal growth.

**Table 6:** The effect of different doses of boron administered to animals on serum Ca, P, Mg, and B levels (*n* = 8)

		Groups					
	Days	Control	B5	B10	B15	*P*	Linear
Ca, mg/dL	0	10,7 ± 0,3	10,4 ± 0,3	10,7 ± 0,3	10,6 ± 0,2	> 0.05	> 0.05
	30	10,3 ± 0,3	10,5 ± 0,2	10,9 ± 0,3	11,2 ± 0,4	> 0.05	0.021
	60	10,2 ± 0,4	10,6 ± 0,4	11,2 ± 0,3	11,1 ± 0,3	> 0.05	0.042
	90	10,2 ± 0,3^b^	10,4 ± 0,2^ab^	11,1 ± 0,4^ab^	11,5 ± 0,4^a^	0.030	0.004
	RM	> 0.05	> 0.05	> 0.05	> 0.05		
	Two-Way RM						
	Time	> 0.05					
	Group	0.004					
	TimeXGroup	> 0.05					
P, mg/dL	0	5,9 ± 0,1	5,9 ± 0,1	5,9 ± 0,1	6,1 ± 0,2	> 0.05	> 0.05
	30	5,9 ± 0,2	5,9 ± 0,1	5,8 ± 0,1	5,9 ± 0,2	> 0.05	> 0.05
	60	5,9 ± 0,2	5,8 ± 0,1	5,9 ± 0,2	5,9 ± 0,2	> 0.05	> 0.05
	90	5,9 ± 0,2	5,9 ± 0,1	5,8 ± 0,2	5,9 ± 0,2	> 0.05	> 0.05
	RM	> 0.05	> 0.05	> 0.05	> 0.05		
	Two-Way RM						
	Time	> 0.05					
	Group	> 0.05					
	TimeXGroup	> 0.05					
Mg, mg/dL	0	1,9 ± 001	1,9 ± 0,1	1,9 ± 0,1	1,9 ± 0,1	> 0.05	> 0.05
	30	1,9 ± 0,1	1,9 ± 0,1	1,9 ± 0,1	1,9 ± 0,1	> 0.05	> 0.05
	60	1,8 ± 0,1	1,9 ± 0,1	1,8 ± 0,1	1,8 ± 0,1	> 0.05	> 0.05
	90	1,8 ± 0,1	1,8 ± 0,1	1,8 ± 001	1,8 ± 001	> 0.05	> 0.05
	RM	> 0.05	> 0.05	> 0.05	> 0.05		
	Two-Way RM						
	Time	> 0.05					
	Group	> 0.05					
	TimeXGroup	> 0.05					
B, mg/L	0	0,026 ± 0,0015	0,025 ± 0,001^D^	0,026 ± 0,0013^C^	0,025 ± 0,001^C^	> 0.05	> 0.05
	30	0,026 ± 0,0017^d^	0,040 ± 0,001^c, C^	0,06 ± 0,0013^b, B^	0,073 ± 0,001^a^,^B^	< 0.001	< 0.001
	60	0,026 ± 0,0012^d^	0,050 ± 0,001^c, B^	0,071 ± 0,0010^b, A^	0,097 ± 0,001^a, A^	< 0.001	< 0.001
	90	0,026 ± 0,0012^c^	0,056 ± 0,011^b, A^	0,075 ± 0,0011^b, A^	0,132 ± 0,014^a, A^	< 0.001	< 0.001
	RM	> 0.05^**^	< 0.001	< 0.001	< 0.001		
	Two-Way RM						
	Time	< 0.001					
	Group	< 0.001					
	TimeXGroup	< 0.001					

The data are presented as mean ± standard error

Data were analyzed using a two-way repeated-measures ANOVA to evaluate the effects of group, time, and their interaction (LSD, *p* < 0.05)

Different lowercase letters (a-d) above the groups indicate significant differences between the means within the same row

Differences between measurement times were analyzed using repeated measures (RM) analysis

Different capital letters (A-D) above the groups indicate significant differences between the means in the same column

** Greenhouse-Geisser

- For Ca, only the group factor was found to be significant (*p* = 0.004). No significant effects of the main factors or their interactions were observed for the other minerals

Serum phosphorus (P) and magnesium (Mg) levels did not show any significant changes across different measurement times between groups (*P* > 0.05, Table 6). Similarly, serum calcium (Ca) levels did not exhibit significant changes until day 60. However, on day 90, serum Ca levels in the B15 group were found to be significantly higher compared to the control group (*P* = 0.03). Serum Ca levels demonstrated a linear increase in relation to dosage on days 30 (*P* = 0.021), 60 (*P* = 0.042), and 90 (*P* = 0.004). Serum boron (B) levels showed a linear increase proportional to the dosage (*P* < 0.001). In all groups except the control group, serum boron levels increased over time with boron supplementation (*P* < 0.001).

**Table 7:** The effects of different doses of boron on serum cortisol, Parathyroid Hormone (PTH), Calcitonin, Osteocalcin, and Alkaline Phosphatase (ALP) levels (*n* = 8)

		Groups					
	Days	Control	B5	B10	B15	*P*	Linear
Cortisol, µg/dL	0	2,2 ± 0,3	2,1 ± 0,2	2,1 ± 0,2	2,2 ± 0,2	> 0.05	> 0.05
	30	2,2 ± 0,2	2,2 ± 0,2	2,4 ± 0,1	2,4 ± 0,2	> 0.05	> 0.05
	60	2,2 ± 0,3	2,4 ± 0,1	2,6 ± 0,1	2,7 ± 0,1	> 0.05	0,026
	90	2,0 ± 0,1^b^	2,5 ± 0,2^ab^	2,7 ± 0,1^a^	2,8 ± 0,2^a^	0,004	< 0.001
	RM	> 0.05	> 0.05	> 0.05	> 0.05		
	Two-Way RM						
	Time	> 0.05					
	Group	0,016					
	TimeXGroup	> 0.05					
PTH, pg/ml	0	36,0 ± 1,9	36 ± 1,5	36,8 ± 2,0	36,6 ± 2,4	> 0.05	> 0.05
	30	36,4 ± 2,2	35,9 ± 2,2	37,4 ± 1,5	37,1 ± 1,7	> 0.05	> 0.05
	60	36,5 ± 1,8	35,6 ± 1,8	35 ± 2,4	36,3 ± 2,2	> 0.05	> 0.05
	90	35,4 ± 1,7	36,5 ± 2,5	36,8 ± 1,9	36,6 ± 2,3	> 0.05	> 0.05
	RM	> 0.05	> 0.05**	> 0.05	> 0.05		
	Two-Way RM						
	Time	> 0.05					
	Group	> 0.05					
	TimeXGroup	> 0.05					
Calcitonin, ng/ml	0	141,1 ± 5,2	140,5 ± 3,9	140,8 ± 4,1^D^	140,9 ± 3,5^D^	> 0.05	> 0.05
	30	142,1 ± 2,7^b^	141,3 ± 2,6^b^	150,7 ± 3,6^b, C^	163,4 ± 2,1^a, C^	< 0.001	< 0.001
	60	140,3 ± 4,8^b^	146,3 ± 3,7^b^	179,2 ± 9,5^a, B^	182,1 ± 6,9^a, B^	< 0.001	< 0.001
	90	142,0 ± 11,6^b^	171,6 ± 13^b^	215,7 ± 7,1^a, A^	233,8 ± 4,7^a, A^	< 0.001	< 0.001
	RM	> 0.05**	> 0.05**	< 0.001	< 0.001		
	Two-Way RM						
	Time	< 0.001					
	Group	< 0.001					
	TimeXGroup	< 0.001					
Osteocalcin, ng/ml	0	10,9 ± 0,5	11,1 ± 0,7^C^	11,0 ± 0,3^C^	11,0 ± 0,5^D^	> 0.05	> 0.05
	30	11,1 ± 0,5	11,2 ± 0,7^C^	12 ± 0,7^BC^	12,2 ± 0,4^C^	> 0.05	> 0.05
	60	12,6 ± 0,8	13,8 ± 0,8^B^	13,7 ± 0,7^B^	15,0 ± 0,5^B^	> 0.05	0,033
	90	11,8 ± 0,5^c^	17,2 ± 0,8^b, A^	17,2 ± 0,8^b, A^	20,6 ± 0,8^a, A^	< 0.001	< 0.001
	RM	> 0.05**	< 0.001	< 0.001	< 0.001		
	Two-Way RM						
	Time	< 0.001					
	Group	< 0.001					
	TimeXGroup	< 0.001					
ALP, U/L	0	202,6 ± 7,8	206,6 ± 6,1	204,3 ± 7,3	200,8 ± 6,3	> 0.05	> 0.05
	30	195,9 ± 9,3	196 ± 5,8	194,3 ± 6,6	195,6 ± 6,7	> 0.05	> 0.05
	60	200,1 ± 10,2	200,4 ± 6,9	190,0 ± 9,7	192,9 ± 9,0	> 0.05	> 0.05
	90	205,3 ± 5,7	192,0 ± 7,3	187,9 ± 8,1	186,4 ± 8,1	> 0.05	> 0.05
	RM	> 0.05	> 0.05	> 0.05	> 0.05		
	Two-Way RM						
	Time	> 0.05					
	Group	> 0.05					
	TimeXGroup	> 0.05					

The data are presented as mean ± standard error

Data were analyzed using a two-way repeated-measures ANOVA to evaluate the effects of group, time, and their interaction (LSD, *p* < 0.05)

Different lowercase letters (a-d) above the groups indicate significant differences between the means within the same row

Differences between measurement times were analyzed using repeated measures (RM) analysis

Different capital letters (A-D) above the groups indicate significant differences between the means in the same column

** Greenhouse-Geisser

- Both the main effects and the interaction effect were significant for osteocalcin and calcitonin (*p* < 0.001)

Serum cortisol levels were measured as a biomarker of metabolic and stress response. A significant increase was observed in the B10 and B15 groups on day 90 compared to the control group (*P* = 0.004), and cortisol levels increased in a dose-dependent manner over time (*P* < 0.001). Although cortisol levels increased in the higher-dose groups, all values remained within physiological reference ranges, suggesting that the observed increase may reflect metabolic adaptation rather than a stress response. Serum PTH levels did not show any significant differences between the groups or across different measurement times. Calcitonin levels showed a dose-dependent increase in the B10 and B15 groups across all time points after baseline (*P* < 0.001), with a significant difference on day 30 for the B15 group (*P* < 0.001). Except for the baseline measurement, calcitonin levels showed a dose-dependent increase at all time points (*P* < 0.001). Additionally, calcitonin levels significantly increased over time in the B10 and B15 groups (*P* < 0.001). Osteocalcin levels were significantly higher in the B15 group on day 90 compared to control (*P* < 0.001), with a dose-dependent linear increase across the study period (*P* < 0.001). These results indicate that boron supplementation positively affects bone formation markers. Throughout the study, serum ALP levels did not differ significantly among the groups across different measurement times (*P* > 0.05, Table 7). Similarly, no statistical differences were detected in these parameters based on dose (*P* > 0.05).

**Table 8:** The Effect of Different Doses of Boron on Serum Vitamin D_3_ Levels in Animals (*n* = 8)

		Groups					
	Days	Control	B5	B10	B15	*P*	Linear
Vit D_3_, ng/ml	0	41,3 ± 2,5	41,2 ± 2,1^B^	40,8 ± 1,6^B^	40,3 ± 0,9^B^	> 0.05	> 0.05
	30	41,9 ± 1,4	41,4 ± 1,8^AB^	41,8 ± 1,6^B^	42,0 ± 1,8^AB^	> 0.05	> 0.05
	60	44,5 ± 3,1	46,0 ± 2,7^AB^	46,5 ± 3,9^B^	45,7 ± 3,0^AB^	> 0.05	> 0.05
	90	45,6 ± 4,0	51,3 ± 3,4^A^	52,7 ± 3,2^A^	52,4 ± 2,4^A^	> 0.05	> 0.05
	RM	> 0.05**	0,028	0,004**	0,001		
	Two-Way RM						
	Time	< 0.001					
	Group	> 0.05					
	TimeXGroup	> 0.05					

The data are presented as mean ± standard error

Data were analyzed using a two-way repeated-measures ANOVA to evaluate the effects of group, time, and their interaction (LSD, *p* < 0.05)

Differences between measurement times were analyzed using repeated measures (RM) analysis

Different capital letters (A-D) above the groups indicate significant differences between the means in the same column

** Greenhouse-Geisser

- Vitamin D levels varied significantly over time (*p* < 0.001), whereas no significant effects of group or interaction were observed

No significant differences were observed between groups in terms of Vitamin D3 levels based on dose (*P* > 0.05). However, in the supplemented groups, Vitamin D3 levels on day 90 were significantly higher than baseline (*P* < 0.05), suggesting a potential interaction of boron with calcium and bone metabolism.

## Discussion

In horses, a successful racing career and high performance during adulthood depend largely on adequate and balanced nutrition during the growth period. While rations must meet requirements for energy, protein, and major minerals (Ca, P, Mg), the inclusion of functional trace elements such as boron may further support optimal development. In the present study, the significant Group × Time interaction observed in metacarpal diameter indicates that boron supplementation modulates skeletal development over time without altering the overall physiological growth pattern.

Boron is a biologically active trace element involved in mineral metabolism, endocrine regulation, and bone development. It has been shown to influence calcium and magnesium absorption, interact with vitamin D-dependent pathways, and enhance osteoblastic activity, thereby contributing to bone mineralization and overall physiological function.

Although numerous studies have investigated the effects of boron in poultry, rodents, and ruminants, there is limited information regarding its role in non-ruminant species such as horses. In this context, the present findings provide evidence on the effects of boron supplementation in Purebred Arabian foals, particularly in terms of growth performance and bone-related parameters. Understanding these effects may contribute to the development of optimized feeding strategies aimed at improving skeletal health and performance in horses.

### Performance Parameters

When comparing the live weight gains among the experimental groups (Table 4), the highest final live weight values were observed in the B15 (288.4 kg) and B10 (288.1 kg) groups, followed by the B5 (284.5 kg) and control groups (284.5 kg). Although these differences were not statistically significant (*P* > 0.05), there was an approximately 8% improvement in the B15 and B10 groups compared to the control and B5 groups.

When examining the daily live weight gains in the groups (Table 4), the highest average daily live weight gain during the experiment was found in the B15 group (567.7 g), followed by the B10 group (543.3 g), the B5 group (512.2 g), and the control group (502.2 g) (*P* = 0.013). As shown in Table 4 there is a linear increase in live weight gain depending on the dose in the groups. Compared to the control group, this increase was approximately 2% in the B5 group, 9% in the B10 group, and 13% in the B15 group.

Feed intake was similar across all groups (Table 4), and no statistically significant differences were found between the groups (*P* > 0.05).

The most efficient FCR during the experiment was observed in the B15 group (17.0), followed by the B10 group (17.9), the control group (19.2), and the B5 group (19.3) (*P* = 0.038) (Table 4). As observed, a linear decrease (improvement) in feed efficiency was seen with boron supplementation, especially in the B15 and B10 groups, in a dose-dependent manner. Compared to the control and B5 groups, this decrease was approximately 5% in the B10 group and 10% in the B15 group. Similarly, compared to the B10 group, the B15 group showed a decrease (improvement) of approximately 5%. These results suggest that boron supplementation may enhance feed utilization efficiency, potentially by influencing enzymatic activity in the digestive system and energy metabolism, and by improving mineral availability relevant to skeletal development.

Regarding morphometric measurements (Table 5), while no significant main effect of the group was observed for metacarpal bone diameter (*P* > 0.05), a highly significant time × group interaction was detected (*P* < 0.001). This indicates that the rate of increase in metacarpal diameter followed a different developmental trajectory in boron-supplemented foals compared to the control group. Similarly, withers height and chest circumference showed significant physiological increases over time (*P* < 0.001), although group-wise differences did not reach statistical significance (*P* > 0.05).

As shown in Table 4 the study demonstrated that the addition of boron resulted in significant improvements in DWG and FCR in a dose-dependent manner, with the most effective dose being 15 mg/day per animal. This positive effect could be attributed to boron’s regulatory role in the digestive system [26], its involvement in the structure of many enzymes in metabolism, its effects on energy metabolism [27], and its positive effects on bone [10–14, 18–28] and mineral metabolism [6]. The positive effects of boron on daily weight gain and FCR in this study are in line with its reported roles in mineral metabolism and bone development. Boron interacts with calcium, phosphorus, and magnesium metabolism, and may modulate vitamin D and osteoblastic activity, contributing to improved growth performance.

Comparisons with other species show mixed results. In broilers, Rossi et al. [29] reported that high boron doses (320 mg/kg) reduced feed intake and live weight, but improved feed efficiency [29]. Fassani et al. [15] observed that 30 ppm boron increased feed efficiency while slightly reducing feed intake [15]. Conversely, some studies found no effect of dietary boron on growth performance in poultry at lower doses [16, 17], highlighting species-specific responses.

In another study by Eren and Uyanık, [16], the addition of 400 ppm boric acid to the diets of laying hens had a negative impact on live weight, feed intake, and egg production, while feed efficiency was not affected [16]. Another study by Yıldız et al. [17] reported no statistical differences between control and experimental groups in terms of average live weight, live weight gain, and feed efficiency throughout the study period [17]. In contrast, in a study by Elhashmi et al. [30] conducted on ostriches, boron supplementation positively affected the growth and development of the tibia, significantly increasing the cortical thickness of the tibia [30]. The variability in results across studies is likely due to differences in species, environmental conditions, management practices, and the form and dose of boron used. Importantly, while these studies provide valuable insights, the present study contributes novel data by evaluating boron supplementation specifically in foals, a non-ruminant species with unique mineral metabolism and growth patterns. The variation in results across species may be attributed to differences in gastrointestinal physiology; specifically, as hindgut fermenters, horses may exhibit unique boron absorption kinetics and interactions with the large colon’s microbial environment compared to the monogastric or avian models cited.

The results of the present study provide novel insights for equine nutrition, demonstrating that boron supplementation at 10–15 mg/day may support growth performance and feed efficiency in young Arabian foals without adverse effects. These findings emphasize the potential role of boron in early life nutrition strategies aimed at optimizing skeletal development and subsequent athletic performance. [31, 32]

### Serum Ca, P, Mg, and B Levels

When examining the serum levels of Ca, P, Mg, and B (Table 6), it was observed that serum levels of P and Mg did not change significantly between groups at different measurement times (*P* > 0.05). Similarly, no significant changes were observed in serum Ca levels up until day 60. However, by day 90, serum Ca levels in the B15 group were significantly higher than in the control group (*P* = 0.03), with a linear increase observed across other experimental groups depending on the boron dose. Serum boron levels also increased linearly in a dose-dependent manner (*P* < 0.001), and in all groups except the control, boron supplementation led to an increase in serum B levels over time (*P* < 0.001). The highest serum B concentration was detected in the B15 group, followed by B10, B5, and control groups. The stability of serum P and Mg levels suggests that boron supplementation, at the doses provided, enhances calcium availability without disrupting the overall mineral homeostasis.

Boron is known to modulate the absorption and metabolism of calcium, phosphorus, magnesium, and vitamin D, supporting mineral homeostasis and bone health. Boron deficiency can lead to mineral imbalances and decreased absorption, altering plasma concentrations [6–33]. Boron has been shown to increase ionized calcium in the blood, enhance vitamin D3 metabolism, and modulate osteoblastic activity, thereby promoting mineral deposition in bone [34, 35] and mitigate the negative effects of magnesium deficiency in chicks by restoring Ca and Mg levels [27]. Similarly, in calves, boron increased serum calcium in a dose-dependent manner [29]. Improvements in serum Ca and Mg concentrations have also been reported in cows [36] and buffaloes [37].In horses specifically, assessing mineral concentrations is critical for growth and future athletic performance [31, 32]. Although data in equines are limited, these findings suggest that boron supplementation may enhance calcium availability and support skeletal mineralization during critical growth periods [32–36, 38, 39].

Boron has also been reported to stimulate osteoblastic activity by modulating Ca²⁺ flow through L-type channels and Na⁺/K⁺-ATPase, enhancing osteoblast proliferation and differentiation [40, 41]. In sheep, dietary boron increased intestinal Ca absorption by 63% compared to controls [26].

However, the effect of boron on serum Mg and P levels is inconsistent; some studies report a positive effect by Kurtoglu et al. [42, 43], while others found that boron supplementation had no effect on these properties by Basoglu et al., [44]; Eren et al., [37]; Bozkurt et al., [45] [37, 44, 45].

The higher serum Ca observed with boron supplementation may be attributed to its interaction with microsomal enzymes, improving vitamin D3 status and enhancing intestinal Ca absorption [10–14, 18–28].

As shown in the Table 6, as the daily amount of boron given to the animals increased, there was a linear increase in serum boron levels. Many studies have reported similar findings, supporting our results, where an increase in the amount of boron added to the diet al.so leads to an increase in serum boron [46–48].

Taken together, these results provide evidence that boron supplementation in foals can positively influence calcium metabolism and mineral status, which is likely to have implications for growth and future skeletal performance. This highlights the importance of considering boron as a strategic micronutrient in equine growth nutrition.

These mineral and hormonal findings can be integrated into a coherent physiological framework. The concurrent stabilization of PTH levels alongside significantly increased serum calcium suggests that boron may enhance calcium utilization or absorption efficiency without inducing a compensatory hyperparathyroid response. Furthermore, the moderate increase in cortisol observed at day 90 likely reflects a metabolic adaptation to accelerated growth and altered mineral flux rather than a typical stress response, as evidenced by the optimal feed conversion and growth parameters maintained throughout the study. May reflect increased metabolic activity associated with growth; however, this interpretation should be approached cautiously, as cortisol is also a well-established stress biomarker.

### Serum Cortisol, Parathyroid Hormone, Calcitonin, Osteocalcin, and ALP Levels

When examining serum cortisol levels (Table 7), it was found that at the end of the study, cortisol levels were significantly higher in the B10 and B15 groups compared to the control group. Additionally, cortisol levels showed a linear increase with respect to the dose. Cortisol is known to influence calcium and phosphate metabolism indirectly by modulating bone turnover and mineral absorption, which may contribute to the observed effects of boron on performance [1–33]. Importantly, all cortisol values remained within the established physiological reference ranges for growing foals, and no clinical or behavioral signs of stress were observed throughout the experimental period. The fact that no decrease in feed intake or growth performance was observed further supports the notion that this increase in cortisol is a physiological response to increased metabolic activity rather than a stress-related condition.

Regarding serum parathyroid hormone (PTH) levels (Table 7), no significant increase was observed in either dose-dependent or time-dependent measurements. However, contrary to our findings, Sharma et al. [38] reported that in buffaloes receiving boron supplementation, the PTH levels were lower in the boron-supplemented group compared to the control group. Similarly, Ismail [49] reported a significant decrease in PTH levels in rats that received boron supplementation [49]. Another study by Sing et al. [8] also found that boron supplementation did not affect serum PTH levels [8]. PTH is a key regulator of calcium and phosphate homeostasis, and boron’s modulatory role may depend on species-specific vitamin D metabolism and mineral status, explaining the variable results [35].

In terms of serum calcitonin levels (Table 7), on the 30th day, the B15 group had higher calcitonin levels than the other groups, while on days 60 and 90, the B10 and B15 groups had higher calcitonin levels than the other groups. Except for the baseline measurement, all subsequent measurements showed a dose-dependent increase in calcitonin levels. Calcitonin levels significantly increased over time in the B10 and B15 groups. Supporting our findings, a study by Ismail [49] also found that boron supplementation increased calcitonin levels in rats [49]. However, another study by Sing et al. [8] on calves reported no effect of boron supplementation on serum calcitonin levels [8].

The increase in calcitonin levels observed in this study can be explained by elevated serum calcium concentrations, as calcitonin secretion is stimulated in response to increased calcium levels. This supports the hypothesis that boron enhances calcium absorption and subsequently modulates endocrine responses involved in bone metabolism.

Serum osteocalcin levels exhibited a statistically significant difference (*P* < 0.001) only in the measurements taken on the 90th day. Additionally, a linear dose-dependent increase in osteocalcin levels was observed (*P* < 0.001). Except for the control group, all other groups showed a time-dependent increase in osteocalcin levels with boron supplementation (*P* < 0.001). Osteocalcin, synthesized by osteoblasts, is crucial for bone matrix formation and hydroxyapatite binding. Boron supplementation appears to stimulate osteoblastic activity and osteocalcin expression, likely through its role in enhancing calcium and phosphate availability and vitamin D metabolism [33–35]. For instance, Ying et al. [50] reported a significant increase in osteocalcin levels in humans receiving boron supplementation [50]. Similarly, study by Hakki et al. [51] found that osteocalcin levels significantly increased in the group supplemented with boron in their study on bone health [51]. Additionally, a study in calves found that serum osteocalcin levels increased in a dose-dependent manner with boron supplementation [8]. An in vitro study demonstrated that boron supplementation at up to 1000 ng/mL increased the mRNA expression of genes related to osteoblast function, such as osteocalcin, collagen type I (COL I), and osteopontin (OPN), in MC3T3-E1 cells [52]. However, in contrast to these findings, Armstrong and Spears [53] reported that boron supplementation up to 15 ppm did not affect these parameters in pigs during their reproductive and growth phases [53].

Alkaline Phosphatase (ALP) is an enzyme found in many tissues of the body, particularly in the liver, bones, and digestive system. It helps in breaking down proteins and is used to measure enzyme levels in the blood to diagnose liver damage or bone disorders. Under normal conditions, the activity of these enzymes in the serum is low, but they gradually enter the bloodstream after hepatocellular damage [54]. Throughout the study, no significant differences were observed in serum ALP levels between the groups at different measurement times (Table 7). Similarly, no dose-dependent statistical differences were found in ALP levels. However, at the 90th-day measurements, a tendency towards a decrease in serum ALP levels was observed in the experimental groups compared to the control group, although this difference was not statistically significant. In a study by Ince et al. [55], boron supplementation at various doses for 7 days in animals with induced liver damage significantly decreased serum ALP levels [55]. The lack of a pronounced effect in our study may be due to the horses being housed in a suitable and comfortable environment, and the dosages administered may have been insufficient for larger animals like horses.

### Serum Vitamin D_3_ Levels

When examining the vitamin D_3_ levels among the groups (Table 8), no statistically significant differences were observed. However, a dose-dependent increase was noted in the experimental groups. At the end of the trial, the highest vitamin D_3_ levels were observed in the B10 (52.7) and B15 (52.4) groups, followed by the B5 group (51.3) and the control group (45.6). As shown, there was a linear dose-dependent increase across the groups. Compared to the control group, the experimental groups showed an increase of approximately 13–15%. This increase may be attributed to the interaction of boron with microsomal enzymes that metabolize 25-hydroxyvitamin D3, improving the vitamin D_3_ status in animals and further enhancing calcium absorption from the intestines [10–14, 18–28]. Furthermore, boron may influence the interplay between vitamin D3, PTH, and calcitonin, modulating calcium-phosphate homeostasis and osteoblastic activity, which supports skeletal growth during the critical developmental stage of foals [33–35].

Although several studies in other species have shown that boron supplementation increases vitamin D levels [41], no statistically significant difference was observed in vitamin D3 levels among the groups in the current study (*P* > 0.05). However, the numerical increase observed over time suggests that further investigation with longer periods might be warranted.

### Practical Implications

This study demonstrates that dietary boron supplementation at a dosage of 10–15 mg/day safely and effectively enhances growth performance and bone metabolism in Purebred Arabian foals. Supplementation was associated with increased average daily gain, improved feed conversion efficiency, and elevated serum concentrations of calcium and boron, alongside key osteogenic markers such as osteocalcin and calcitonin, reflecting heightened osteoblastic activity and mineralization.

While metacarpal diameter exhibited a physiological increase across all groups ($*P* < 0.001$), the significant time × group interaction ($*P* < 0.001$) demonstrates that boron supplementation did not merely increase bone diameter but rather modulated the developmental trajectory of the skeleton. These findings suggest that boron exerts a more pronounced effect during rapid-growth phases, facilitating a more synchronized skeletal maturation compared to the control group. Consequently, boron appears to possess a multifactorial mechanism of action, promoting robust skeletal development by optimizing nutrient utilization while simultaneously supporting the endocrine regulation of calcium-phosphate homeostasis.

From a clinical and practical perspective, boron may serve as a strategic micronutrient during the early growth phase to improve developmental efficiency and long-term musculoskeletal health—factors of paramount importance for foals destined for racing or high-performance disciplines. Furthermore, the study underscores species-specific responses in equines, highlighting the necessity for equine-focused nutritional evaluations rather than relying on data extrapolation from other species. Overall, these results support the integration of boron into foal nutritional strategies and provide a foundation for future research to refine optimal dosing and explore synergistic interactions with other essential minerals in equine nutrition.

There are some limitations to the present study that should be acknowledged. First, the sample size (*n* = 8 per group) was relatively small, which may limit the generalizability of the findings to a broader population of horses. Second, the study focused only on male foals; therefore, potential sex-related differences in response to boron supplementation were not evaluated. Finally, although the 90-day period provided significant insights into growth and mineral metabolism, longer-term studies are needed to determine the lifetime effects of boron on bone density and overall health in athletic horses.

## Conclusion

Boron supplementation improved growth performance and influenced selected markers of bone metabolism, particularly serum calcium, osteocalcin, and calcitonin levels, in Purebred Arabian foals. Among the tested doses, 15 mg/day showed the most pronounced effects. While 15 mg/day demonstrated the most pronounced beneficial effects under the conditions of the present study, further research using extended supplementation periods and refined dose ranges is warranted to define optimal boron requirements in horses.

## Data Availability

Dataset and analyses are available from the corresponding author upon reasonable request.

## Declarations

### Animal Welfare

The authors confirm that the ethical policies of the journal, as noted on the journal’s author guidelines page, have been adhered to and the appropriate ethical review committee approval has been received. The authors confirm that they have followed EU standards for the protection of animals used for scientific purposes legislation. This study was approved by the Local Animal Ethics Committee of Inönü University (Decision No: 2021/26-2-13092).

## Abbreviations

B: Boron
P: phosphorus
Mg: Magnesium
Ca: Calcium
PTH: Parathyroid Hormone
ALP: Alkaline Phosphatase
LWG: Live Weight Gain
FCR: Feed Conversion Ratio
DWG: Daily Weight Gain
RPM: Revolutions per Minute
PPM: Parts per Million
ME: Metabolic Energy
mg: Milligram
WHO: World Health Organization
EFSA: European Food Safety Authority
BOREN: National Boron Research Institute(TR)
